# Digital Structured Education With Behavioral Nudge Tools for Adults With Type 2 Diabetes: Multicenter Randomized Controlled Trial

**DOI:** 10.2196/91407

**Published:** 2026-09-01

**Authors:** Yan Lin, Yingchun Zeng, Kaining Chen, Zongcun Chen, Caihua Ye, Ying Zhou, Qiwei Zhou, Chengying Yu, Vivien Xi Wu, Samuel Seidu, Bin Li, Xinjun Jiang

**Affiliations:** 1 School of Nursing, Hainan Medical University, No 3 Xueyuan Road, Longhua district, Haikou, Hainan, China, 86 18789028632; 2 The Second Affiliated Hospital of Hainan Medical University, Haikou, China; 3 National University of Singapore, Singapore, Singapore; 4 Hainan General Hospital, Haikou, China; 5 928th Hospital of PLA Joint Logistics Support Force, Haikou, China; 6 Leicester Diabetes Centre, Leicester, United Kingdom; 7 Leicester General Hospital, Leicester, United Kingdom; 8 Key Laboratory of Emergency and Trauma of Ministry of Education, Engineering Research Center for Hainan Biological Sample Resources of Major Diseases, Key Laboratory of Tropical Cardiovascular Diseases Research of Hainan Province, The First Affiliated Hospital of Hainan Medical University, Haikou, China

**Keywords:** type 2 diabetes, digital structured education, behavioral nudges, self-management behaviors, randomized controlled trial

## Abstract

**Background:**

Digital interventions offer scalable alternatives to traditional face-to-face diabetes education, but often face challenges related to inconsistent clinical effectiveness, and declining user engagement. However, whether a digital structured education program integrated with behavioral nudge tools can improve metabolic, behavioral, and psychological outcomes in adults with type 2 diabetes remains unclear.

**Objective:**

This study aimed to evaluate the effectiveness of a digital structured education program integrated with behavioral nudge tools in improving metabolic, behavioral, and psychological outcomes among adults with type 2 diabetes.

**Methods:**

This multicenter randomized controlled trial was conducted in the endocrinology departments of 4 hospitals in China. Adults with type 2 diabetes were randomly assigned to an intervention group receiving a digital structured education program integrated with behavioral nudge tools (n=146) or a control group receiving standard digital diabetes education (n=147). Assessments were conducted at baseline and 12-week follow-up. The primary outcome was hemoglobin A_1c_ (HbA_1c_) at 12 weeks, adjusted for baseline HbA_1c_, and study center. Secondary outcomes included fasting blood glucose (FBG), weight, BMI, waist circumference, blood pressure, lipid profiles, self-management behaviors, self-efficacy, and habit strength.

**Results:**

Among 293 participants (mean age 49.19, SD 10.02 y), 287 (97.9%) completed follow-up. At 12 weeks, the intervention group demonstrated significantly greater improvements than the control group in HbA_1c_ (adjusted mean difference −0.38%, 95% CI −0.68% to −0.09%; *P=*.01), FBG (adjusted mean difference −0.75, 95% CI −1.27 to −0.44 mmol/L; *P*<.001), weight (adjusted mean difference −0.84, 95% CI −1.61 to −0.07 kg; *P=*.03), BMI (adjusted mean difference −0.38, 95% CI −0.65 to −0.11 kg/m²; *P=*.01), systolic blood pressure (adjusted mean difference −2.71, 95% CI −4.62 to −0.79 mm Hg; *P=*.01), diastolic blood pressure (adjusted mean difference −2.92, 95% CI −4.47 to −1.37 mm Hg; *P*<.001), and total cholesterol (adjusted mean difference −0.27, 95% CI −0.48 to −0.05 mmol/L; *P=*.02). The intervention was also associated with significantly greater improvements in self-management behaviors, self-efficacy, and habit strength (all *P*<.05).

**Conclusions:**

Digital structured education integrated with behavioral nudge tools improved metabolic outcomes and strengthened psychological and behavioral determinants of self-management among adults with type 2 diabetes over a 12-week period. These findings suggest that a digital structured education program integrated with behavioral nudge tools may enhance diabetes self-management beyond standard digital diabetes education. Further studies with longer follow-up and real-world implementation are warranted to evaluate the sustainability, generalizability, and long-term clinical impact of this integrated intervention.

## Introduction

### Background

Type 2 diabetes is a major global public health challenge and one of the fastest-growing chronic diseases. The International Diabetes Federation estimates that 589 million adults aged 20-79 years were living with diabetes in 2024, including approximately 148 million in China, where type 2 diabetes accounts for 90%-95% of cases [1]. Diabetes contributes to more than 3.4 million deaths annually worldwide and generates nearly US $1 trillion in health care expenditures, posing substantial clinical, economic, and societal burdens [1]. In China, the high prevalence of type 2 diabetes and its complications underscores an urgent need for effective and scalable management strategies.

Optimal diabetes management requires maintained glycemic and lipid control, blood pressure regulation, and long-term adherence to self-management behaviors [2,3]. However, control rates for blood glucose, blood pressure, and lipids remain suboptimal in mainland China, partly due to limited access to structured education and insufficient long-term engagement in self-management [4,5]. Improving maintained behavior change remains a central challenge in chronic disease management. Behavior change is increasingly understood through the lens of dual-process theory, which describes maintained 2 interacting systems: a reflective system that supports deliberate, goal-directed decision-making and an automatic system that drives habitual behavior through environmental cues [6-8].

Digital health interventions have emerged as scalable tools for diabetes management, providing remote education, monitoring, and personalized feedback through mobile and web-based platforms [9,10]. Evidence indicates that digital approaches can improve glycemic control, reduce health care usage, and enhance patient empowerment, particularly in resource-limited settings [7,11-13]. However, despite their potential, existing evidence on digital interventions reveals inconsistent findings regarding efficacy, with some studies highlighting challenges in maintaining engagement and a reliance on passive features like reminders rather than comprehensive behavioral strategies [9,14].

### Behavioral Nudges in Digital Diabetes Management

Behavioral nudge tools, grounded in nudge theory, represent an evolving innovation in health behavior change, subtly altering environments or choices to promote healthier decisions without limiting autonomy [15-17]. These tools have been applied in various chronic disease contexts, including obesity and cardiovascular health, showing promise in fostering habits through unconscious cues, such as default options or timely prompts [17,18]. Nevertheless, digital structured education programs integrated with behavioral nudge tools remain underexplored, particularly in China, where adoption is limited and often disconnected from structured educational frameworks.

A digital structured education program integrated with behavioral nudge tools may better address both reflective and automatic drivers of behavior by combining complementary intervention components. Digital education enhances knowledge, self-efficacy, and intentional self-management, while behavioral nudge tools target automatic processes to build habits, such as through gamified prompts or environmental cues that encourage maintained actions like physical activity or medication adherence [19-21]. By simultaneously targeting these complementary behavioral processes, such integrated programs may reduce cognitive load, facilitate maintained behavior change, and potentially achieve greater improvements than standard digital diabetes education alone.

### Knowledge Gap

Despite these synergies, significant gaps persist in the integration of nudge tools with digital education for adults with type 2 diabetes. Limited high-quality evidence from multicenter randomized controlled trials evaluating digital structured education programs integrated with behavioral nudge tools, especially in diverse populations like those in China, hinders understanding of their effectiveness and cultural adaptability [22]. Additionally, there is a scarcity of studies examining how these integrated interventions affect psychological outcomes (eg, habit strength and self-efficacy) alongside metabolic ones, and how they can be scaled equitably amid varying digital literacy levels.

### Study Objective

To address these gaps, we conducted a multicenter randomized controlled trial to evaluate the effectiveness of a digital structured education with behavioral nudge tools (DSE-BN) program compared with standard digital diabetes education developed by the Chinese Diabetes Association among adults with type 2 diabetes in China. Guided by dual-process theory, the DSE-BN program was designed to target both reflective and automatic behavioral processes through the integration of digital structured education and behavioral nudge tools. We hypothesized that, compared with standard digital diabetes education, the DSE-BN program would produce greater improvements in metabolic outcomes (hemoglobin A_1c_ [HbA_1c_], fasting blood glucose [FBG], weight, BMI, blood pressure, and lipid profiles), behavioral outcomes (diabetes self-management behaviors), and psychological outcomes (self-efficacy and habit strength) among adults with type 2 diabetes in China.

## Methods

### Study Design and Setting

This multicenter randomized clinical trial was conducted across 3 tertiary hospitals in Hainan Province, China—Hainan Provincial People’s Hospital and the First and Second Affiliated Hospitals of Hainan Medical University. To ensure adequate recruitment, 928th Hospital was added as an additional study site after trial initiation; no other substantive changes were made to the trial design, outcomes, or analytical plan.

Participants were recruited between March and September 2024 and were randomly assigned in a 1:1 ratio to either the intervention or control group. Follow-up assessments of both benefits and harms were completed at 12 weeks, between June 2024 and December 2025. An interim analysis was conducted midway through the trial to monitor data quality, recruitment progress, and participant safety. Given the low-risk nature of the behavioral education intervention, no formal stopping rules were predefined, and the trial was completed as planned without early termination. Reporting followed the CONSORT (Consolidated Standards of Reporting Trials) checklist.

### Participants

Eligible participants were adults aged 18 to 65 years with type 2 diabetes diagnosed according to the 2020 Chinese guideline for the prevention and treatment of type 2 diabetes mellitus [23], receiving lifestyle interventions alone or combined with oral agents, cognitively and communicatively intact, and able to use a smartphone with guidance. Exclusion criteria included severe diabetes-related complications, mental or cognitive disorders, mobility impairment, recent cancer treatment, or participation in other trials.

The sample size was calculated using PASS (Power Analysis and Sample Size; version 15.0; NCSS) with the tests for 2 means in a multicenter randomized design procedure, using HbA_1c_ as the primary outcome. Assuming a between-group difference of 0.3% and an SD of 0.85% [10], with a 2-sided α of .05 and 80% power, the required sample size was 227 participants. Because the PASS multicenter randomized design procedure requires specification of an intraclass correlation coefficient (ICC), and no empirical estimate of the ICC was available from previous comparable studies, an ICC of 0.10 was adopted as a conservative design assumption, following the recommendation provided in the PASS software documentation, to account for potential within-site correlation. Allowing for a 20% attrition rate, the target sample size was 284 participants. Ultimately, 293 participants were enrolled, exceeding the prespecified target sample size.

### Randomization and Blinding

Participants were randomly allocated in a 1:1 ratio to the intervention or control group using a computer-generated randomization sequence created with SPSS (version 26.0; IBM Corp). Individual-level simple randomization was adopted. Neither block randomization nor stratified randomization by study center was implemented; therefore, allocation block size was not applicable. After eligible participants had been recruited at each study site, an independent researcher centrally assigned participants according to the pregenerated randomization sequence. The randomization process was independent of participant recruitment and outcome assessment, and allocation concealment was maintained throughout the allocation process, thereby minimizing the risk of selection bias across study sites.

Due to the nature of the behavioral intervention, blinding of participants and intervention nurses was not feasible. However, physicians responsible for routine clinical care and outcome assessment, as well as data analysts, were blinded to group allocation to minimize potential performance and detection bias. In addition, study center was included as a covariate in the primary statistical analyses to account for potential between-center differences.

### Intervention

#### Intervention Implementation

Each study site was supported by at least 1 physician and 1 nurse, operating within a centrally coordinated intervention team. Physicians were responsible for clinical diagnosis and participant recruitment, while nurses delivered the intervention and managed informed consent, baseline and follow-up data collection, registration on the self-management support system (SMASS), and ongoing participant support. Study procedures and intervention delivery were coordinated centrally to ensure consistency across sites.

To standardize implementation of the intervention across study sites, all intervention personnel completed uniform, centralized training before study initiation. Training covered intervention content, delivery procedures, use of the SMASS platform, and fidelity requirements. Competency assessments were conducted following training, and only personnel meeting predefined competency criteria were authorized to deliver the intervention. Ongoing supervision and cross-site communication were used to maintain consistency of implementation throughout the study period.

#### Intervention Group

Participants in the intervention group received the DSE-BN program, a nurse-developed, theory-informed digital structured education program incorporating an enhanced educational workflow, shared digital support features, and behavioral nudge tools. Grounded in dual-process theory, the DSE-BN program was designed to influence both reflective and automatic behavioral processes. Reflective behavior change was targeted through structured digital education aimed at improving diabetes-related knowledge and enhancing self-efficacy via progressive goal setting, structured knowledge learning, repetition and review, timely positive feedback, experience sharing, and reminders, while automatic behavioral processes were addressed through subtle environmental modifications using behavioral nudge tools embedded in participants’ everyday environments.

The DSE-BN program comprised two integrated components: (1) a theory-informed digital structured education program incorporating an enhanced educational workflow and shared digital support features and (2) behavioral nudge tools. The digital structured education component was designed to facilitate reflective behavior change through structured educational activities and enhanced educational workflow, whereas the behavioral nudge component targeted automatic behavioral processes by providing environmental cues embedded within participants’ daily living environments. These 2 components were implemented simultaneously throughout the intervention period as complementary elements of a single integrated intervention.

To facilitate comparison, the key components and distinguishing features of the DSE-BN and control interventions are summarized in Table 1.

**Table 1.** Comparison of the key components and distinguishing features of the DSE-BN^a^ and standard digital diabetes education interventions.

Intervention component or feature	Intervention group (DSE-BN)	Control group (standard digital diabetes education)
Intervention overview		
Overall intervention	A nurse-developed, theory-informed digital structured education program integrated with behavioral nudge tools	Standard digital diabetes education based on the Chinese Diabetes Society educational materials
Behavioral theory	Dual-process theory	Not explicitly theory-informed
Primary objective	Promote sustainable diabetes self-management through an enhanced educational workflow, shared digital support features, and environmental behavioral nudges	Deliver standardized diabetes education to improve diabetes-related knowledge and self-management
Educational design		
Educational content	Theory-informed digital structured education	Standard diabetes education based on the Chinese Diabetes Society educational materials
Educational structure	4 structured weekly digital learning modules with an enhanced educational workflow	4 weekly digital educational modules
Delivery platform	SMASS^b^ digital platform	SMASS digital platform
Shared digital support features		
Sequential learning	✓	✓
Discussion area for peer interaction and professional support	✓	✓
Automated learning reminders	✓	✓
Rule-based tailored feedback	✓	✓
Data-driven early-warning alerts (presented using a traffic-light system for metabolic status)	✓	✓
Enhanced educational workflow		
Preparatory questions before each module	✓	—^c^
Review questions after each module	✓	—
Immediate feedback on review questions	✓	—
Behavioral nudge component		
Behavioral nudge tools	✓ (Self-monitoring reminder sticker, portion-control plate, footprint exercise mat, foot-care reminder image, and medication reminder sticker)	—

a DSE-BN: digital structured education with behavioral nudge tools.

b SMASS: self-management support system.

c Not applicable.

### Digital Structured Education

The digital structured education component incorporated structured educational content, an enhanced educational workflow, and shared digital support features delivered individually and remotely through 4 weekly modules via the SMASS platform, enabling participants to complete the program independently in their home environments using their smartphones over 4 consecutive weeks (1 session per week; 9‐25 min per module; total education time approximately 74 min) [11,12,24]. Module content covered diabetes knowledge and self-monitoring, diet management, physical activity and foot care, and regular examinations and follow-up (Table 2). Educational materials included prerecorded videos, interactive quizzes, and learning checkpoints. Modules were completed sequentially, with progression restricted until completion of the current module.

Following completion of baseline assessments, participants commenced the DSE-BN program. Each module followed a standardized learning workflow, beginning with brief preparatory questions before video viewing. Educational videos were presented in a preset order and could not be skipped or fast-forwarded to ensure completion of all learning content. After each module, participants completed review questions and received immediate feedback through encouraging language and supportive visual cues (eg, emojis) to reinforce knowledge acquisition and active learning.

**Table 2.** Components and delivery of the digital structured education with behavioral nudge tools (DSE-BN) program^a^.

Contents of each module	Tools: design rationale and description
Module 1: knowledge about diabetes and self-monitoring	
This module introduces the importance of diabetes management and facilitates patients' self-monitoring. It covers normal blood glucose ranges, hyperglycemia, and hypoglycemia, emphasizing the significance of routine self-monitoring and accurate record-keeping. Patients are encouraged to recognize early warning signs, actively engage in managing their blood glucose levels by self-monitoring.	Self-monitoring reminder sticker^b^: Developed as a reminder sticker to be placed in prominent household locations or near medications, serving to prompt patients while also reinforcing the blood glucose and urine monitoring components of the structured education program.
Module 2: diet management	
Building on module 1, this session highlights the role of weight management in diabetes care. Patients are introduced to different nutrients and calorie, values using visual aids such as food images. Practical exercises include evaluating and calculating daily dietary intake. Patients are encouraged to set personalized dietary goals and develop a structured meal plan tailored to their needs.	Nudge plate^c^: A real, 3-color round plate was developed for home dietary management. The plate adopts a traffic-light scheme, divided into 1 red zone, 2 yellow zones, and 1 green zone. Rim markings correspond directly to the food classification cards used in the structured education program. The green zone is designated for low-calorie, vegetables, the yellow zones for moderate-calorie, foods such as staple grains, lean meat, eggs, poultry, and root vegetables, and the red zone for high-calorie, items such as sugary or fatty foods. Notably, the red zone is half the size of the other colored zones. By incorporating familiar traffic-light cues and linking them with the structured education cards, the plate provides an intuitive visual reminder that encourages patients to increase vegetable consumption, consume moderate portions of staple foods and lean proteins, and limit the intake of high-calorie, foods.
Module 3: physical activity and foot care	
This module reviews the dietary principles discussed previously and emphasizes the importance of regular exercise in diabetes management. Patients are guided to set achievable exercise goals and create individualized activity plans. The session also includes education on foot care, with demonstrations of foot exercises to reduce complications and encouragement to integrate these practices into daily routines. Patients are encouraged to conduct foot care and take exercise.	Footprint exercise mat^b^: Designed as a green footprint exercise mat, with a starting point labeled “6000 steps per day.” Placed at the household entrance, it serves as a daily prompt for physical activity, creating a supportive proximal home microenvironment while reinforcing the exercise component of the structured education program. Foot care reminder image^b^: Developed as a reminder image to be displayed at home, prompting patients to perform daily foot care and providing guidance on proper techniques, while reinforcing the foot care component of the structured education program.
Module 4: regular examinations and follow-up	
The final module revisits the key points of physical activity and foot care, then expands on the importance of regular medical examinations. Patients are educated about appropriate timing, essential laboratory tests, and screening for diabetes-related complications. Medication management is introduced, with a focus on adherence and understanding prescribed treatments. Patients are encouraged to maintain their goals and attend regular follow-up visits with health care providers.	Medication reminder sticker^b^: Developed as a reminder sticker to be placed in the home’s drinking area, serving to prompt patients while reinforcing the medication adherence component of the structured education program.

a Digital structured education was delivered through the self-management support system platform as 1 session per week for 4 consecutive weeks (9-25 minutes per module; total education time approximately 74 minutes). Behavioral nudge tools were introduced at the beginning of the intervention and used continuously in participants’ home environments throughout the 12-week intervention to provide ongoing environmental cues reinforcing healthy self-management behaviors. Refer to the study by Liu et al [24] for the structured education program.

b China Copyright Protection Center. Copyright registration certificate for the work “Self-Management Behavior Illustrations.” Registration No. 国作登字-2024-F-00022150. Beijing: China Copyright Protection Center; 2024 [25].

c Nudge plate is a patent product [26].

Beyond the enhanced educational workflow described above, participants in the DSE-BN group also received the same digital support features available to the control group. These shared digital support features included a discussion area for peer interaction and professional support, automated reminders, rule-based tailored feedback, and data-driven early-warning alerts. Within the discussion area of the SMASS platform, participants could post questions or comments and interact with health care professionals and other participants, thereby facilitating peer interaction and professional support. Automated reminders encouraged timely completion of the learning modules. Rule-based tailored feedback was generated according to participants’ learning progress and self-reported metabolic indicator records uploaded through the SMASS platform and was accompanied by encouraging messages to reinforce self-management behaviors. In addition, participants’ metabolic status was displayed using a traffic-light system (green, yellow, and red) to facilitate timely recognition of abnormal results and encourage appropriate self-management actions.

### Behavioral Nudge Tools

The behavioral nudge component consisted of a set of environmental cueing tools (Table 2), including a self-monitoring reminder sticker, a nudge plate, a footprint exercise mat, a foot care reminder image, and a medication reminder sticker. These tools were designed to subtly encourage healthy behaviors by modifying participants’ everyday environments while preserving individual autonomy.

Standardized instructional videos embedded within the SMASS platform guided participants on how to place and use each behavioral nudge tool appropriately in their homes and daily living environments according to their individual routines. These environmental cues remained in participants’ home environments throughout the intervention period, providing continuous environmental cues to reinforce healthy self-management behaviors.

### Intervention Fidelity

Intervention fidelity was ensured through standardized educational content, centralized training of intervention personnel, restricted module progression, automated reminders, and system-based tracking of learning activities. Engagement with the digital education component was monitored using SMASS system logs. Among the 146 participants allocated to the DSE-BN group, platform records verified that 142 participants completed all 4 educational modules, 1 participant completed 3 modules, and 3 participants completed 1 or fewer modules. Implementation of the behavioral nudge tools was confirmed through participant-submitted photographs of home-based installations reviewed by research nurses. No substantive modifications were made to the DSE-BN program during the study period.

### Control Group

Participants in the control group received standard digital diabetes education delivered through the SMASS platform, with educational content developed by the study team based on educational materials from the Chinese Diabetes Association. The control program consisted of 4 weekly sessions (15‐30 min per session; total approximately 103 min) covering diabetes basics, pharmacotherapy, glucose monitoring, complications, and lifestyle management. WeChat (Tencent Holdings Ltd) was used only for routine communication and follow-up management and was not part of the intervention delivery.

Similar to the DSE-BN group, participants completed the educational modules through the SMASS platform and had access to the same shared digital support features, including sequential learning, a discussion area for interaction with health care professionals and other participants, automated reminders, rule-based tailored feedback, and data-driven early-warning alerts throughout the intervention period. However, unlike the DSE-BN program, the control intervention did not include the enhanced educational workflow, including preparatory questions before video learning and postmodule review questions with immediate feedback, or home-based behavioral nudge tools designed to provide home-based environmental cues for behavior change.

Among the 147 participants in the control group, completion of the 4 digital education modules was tracked through the platform. Platform records indicated that 113 participants completed all 4 modules, 25 completed 3 modules, 6 completed 2 modules, and 3 completed 1 or fewer modules.

All participants in both groups continued to receive routine diabetes care, including outpatient visits, laboratory monitoring, and pharmacological treatment as prescribed by their physicians.

### Outcome Measures

All metabolic, psychosocial, and behavioral outcome data were collected at baseline and at the 12-week follow-up (March 2024 to December 2025). The primary outcome was HbA_1c_ level at 12 weeks. Secondary metabolic outcomes of blood lipid profiles (total cholesterol [TC], triglycerides, high-density lipoprotein cholesterol [HDL], and low-density lipoprotein cholesterol [LDL]) were extracted from medical records at each study site. Additional secondary outcomes, including FBG, blood pressure, weight, waist circumference (WC), BMI, self-efficacy, habit strength, self-management behaviors, and other outcomes of medications, were assessed and recorded by trained research nurses using reliable and validated instruments.

Diabetes self-efficacy was assessed using the validated Chinese version of the Self-Efficacy for Diabetes (C-SED) scale [27]. The original scale was developed by Lorig and colleagues [28] in the United States and was subsequently translated and culturally adapted into Chinese by Wei et al [27]. The C-SED comprises 9 items covering 4 domains: diet, physical activity, blood glucose management, and disease control. Each item is rated on a 5-point Likert scale, ranging from 1 (“not at all confident”) to 5 (“completely confident”). The mean score ranges from 1 to 5, with higher scores indicating greater diabetes self-efficacy. The Chinese version has demonstrated good reliability in previous studies, and the Cronbach α in this study was 0.87.

Habit strength was assessed using the Self-Report Habit Index (SRHI) [29,30]. The original SRHI consists of standardized descriptive statements that can be adapted to specific behavioral contexts (eg, “XXX is something I do automatically”). In this study, the SRHI was adapted to assess 5 diabetes self-management behaviors: dietary management, physical activity, blood glucose monitoring, foot care, and medication adherence. Following contextual adaptation and expert review, a 20-item questionnaire was developed, comprising 4 items for each behavior. All items were rated on a 5-point Likert scale, ranging from 1 (“strongly disagree”) to 5 (“strongly agree”). Total scores range from 20 to 100, with higher scores indicating stronger habit strength [30]. The Cronbach α in this study was 0.94.

Diabetes self-management behaviors were assessed using the validated Chinese version of the Summary of Diabetes Self-Care Activities questionnaire [31]. The questionnaire comprises 11 items across 6 domains: general diet (2 items), specific diet (2 items), physical activity (2 items), blood glucose monitoring (2 items), foot care (2 items), and medication adherence (1 item). Each item assesses the number of days per week (0‐7 d) on which the corresponding self-management behavior was performed. Total scores range from 0 to 77, with higher scores indicating better diabetes self-management behaviors. Item 4 is reverse scored, whereas all remaining items are scored in the positive direction. The Cronbach α in this study was 0.78.

Harms were defined as severe hypoglycemia, characterized by events requiring assistance from another person or medical attention. These events were assessed systematically at the 12-week follow-up visit through structured participant interviews and review of medical records, and supplemented by nonsystematic participant self-reports during the study period. All reported events were documented and verified by site investigators.

Demographic and clinical characteristics were collected using a self-designed questionnaire. Information collected included age, sex, ethnicity, medical insurance, educational background, employment status, marital status, cohabitation group, individual monthly income, smoking status, alcohol consumption, and electronic health management. Clinical characteristics included type 2 diabetes duration, type 2 diabetes medication duration, types of medications, and diabetes-related complications.

### Quality Control

To ensure consistency and methodological rigor across study sites, standardized operating procedures were used to guide participant recruitment, intervention delivery, data collection, and follow-up. All study personnel received uniform training before study initiation, and adherence to the study protocol was reinforced throughout the trial.

Intervention fidelity was supported through system-based controls embedded within the SMASS platform, including restricted module progression, automated reminders, and engagement prompts (eg, pre- and postmodule quizzes and timed pauses). Nurses monitored participants’ engagement and progress on a weekly basis, provided reminders as needed, and assisted with scheduling follow-up assessments. To minimize potential contamination between study groups, intervention materials and behavioral nudge tools were accessible only to participants randomized to the intervention group, and participants in the intervention and control groups were followed up at separate time points.

Quality assurance procedures were implemented at both the site and central levels. Monthly site monitoring was conducted to assess protocol adherence and resolve implementation issues. Data accuracy and completeness were ensured through double data entry and independent expert review. Any discrepancies were resolved by consensus. When necessary, original source data were verified by contacting participants, and records were corrected accordingly.

### Ethical Considerations

The trial was registered with the Chinese Clinical Trial Registry (ChiCTR2400082373) on March 27, 2024. The study protocol is available at the registry website, and deidentified study data are available from the corresponding author upon reasonable request. Ethical approval was granted by the Ethics Committee of Hainan Medical University (HYLL-2023‐461), and the study was conducted in accordance with the Declaration of Helsinki. Before enrollment, all participants received detailed information regarding the study objectives, study procedures, potential risks and benefits, and measures to protect their privacy and confidentiality. Written informed consent was obtained from all participants before participation, and participants were informed of their right to withdraw from the study at any time without affecting their medical care.

To protect participants’ privacy and confidentiality, all study data were deidentified before analysis and securely stored, with access restricted to authorized research personnel only. Participants received free access to the SMASS platform throughout the study and could consult the research team at no cost during the intervention period. Participants in the intervention group also received the behavioral nudge tools free of charge, including a self-monitoring reminder sticker, a nudge plate, a footprint exercise mat, a foot care reminder image, and a medication reminder sticker. No monetary compensation was provided for participation in the study.

### Statistical Analysis

Analyses were conducted using SPSS (version 27.0) under the intention-to-treat principle. At the 12-week follow-up, outcome data were missing for 6 of the 293 (2%) participants. Missing values were observed for weight, BMI, WC, FBG, HbA_1c_, lipid profiles, self-efficacy, diabetes self-management behaviors, and habit strength. These missing outcome data were handled using multiple imputation before the primary analyses. A total of 5 imputed datasets were generated via a Markov chain Monte Carlo approach with 10 iterations per dataset. Predictive mean matching was applied for continuous variables, and logistic regression was used for categorical variables. Final estimates were derived by pooling results across datasets according to Rubin’s rules.

Baseline characteristics were summarized using means and SDs for normally distributed continuous variables or medians (IQRs) for nonnormally distributed variables, as appropriate, and numbers (percentages) for categorical variables. Between-group comparisons of baseline characteristics were conducted using independent-samples *t* tests, chi-square tests, Fisher exact tests, or Mann-Whitney *U* tests, as appropriate.

The primary analysis compared HbA_1c_ at 12 weeks between the intervention and control groups using ANCOVA, with adjustment for baseline HbA_1c_ and study center as a fixed effect. The same baseline- and center-adjusted ANCOVA approach was applied to secondary outcomes. Sensitivity analyses for both primary and secondary outcomes at 12 weeks were conducted using ANCOVA with adjustment for baseline values only.

Between-group comparisons of other outcomes, including achievement of an HbA_1c_ reduction ≥0.5% and differences in medication use at 12 weeks, were performed using chi-square tests or Mann-Whitney *U* tests, as appropriate. Within-group comparisons of primary and secondary outcomes between baseline and 12 weeks were conducted using paired *t* tests or Wilcoxon signed-rank tests, as appropriate. All tests were 2-sided, with a *P* value <.05 considered statistically significant.

## Results

### Overview

**Figure 1.**
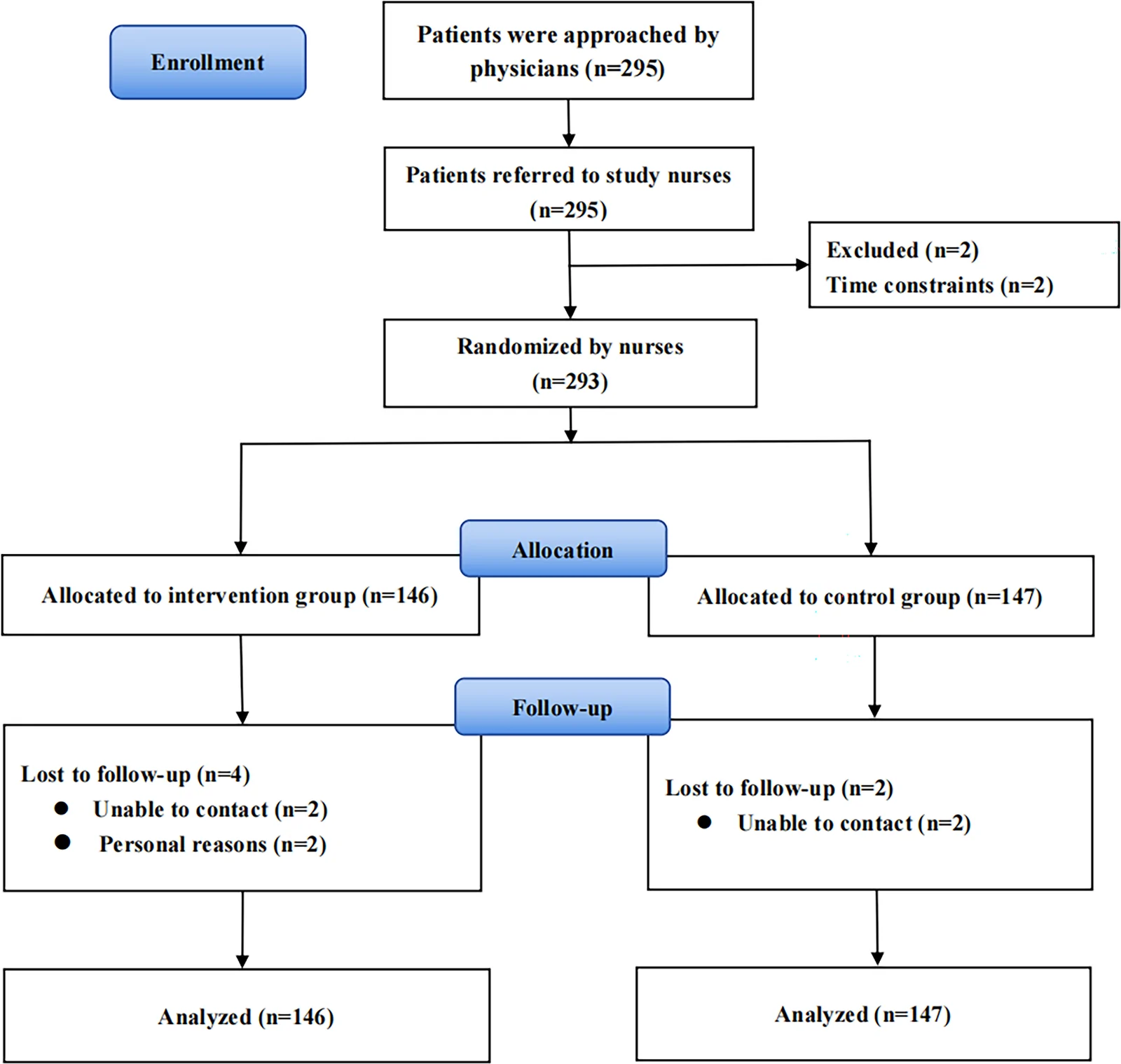
The study flowchart.

A total of 295 participants met the inclusion criteria, of whom 293 (99.3%) consented and completed baseline data collection. Participants were randomized to the intervention group receiving digital structured education with nudging tools (n=146) or the control group receiving standard digital health education (n=147). Moreover, 4 participants in the intervention group and 2 in the control group were lost to follow-up, each completing fewer than 2 modules (Figure 1).

### Baseline Characteristics

The 293 participants had a mean age of 49.19 (SD 10.02) years; 191 (65.2%) were male, and 281 (95.9%) were of Han ethnicity. Most participants were married (n=254, 86.7%), employed (n=211, 72%), and had completed at least junior high school education (n=157, 53.6%). About two-thirds (n=196, 66.9%) were covered by urban employee or urban-rural resident medical insurance. The mean duration of type 2 diabetes was 3.94 (SD 4.39) years, with a mean treatment duration of 3.36 (SD 4.32) years; 208 (71%) reported no diabetes-related complications. Overall, 184 (62.8%) had not previously used electronic devices for disease management. Baseline characteristics of the 2 groups are presented in Table 3.

**Table 3.** Characteristics of patients with type 2 diabetes mellitus (N=293).

Characteristics	IG^a^ (n=146)	CG^b^ (n=147)	*t* test (*df*) or chi-square (*df*)	*P* value
Age (y), mean (SD)	48.14 (10.25)	50.22 (9.72)	−1.784 (291)^c^	.08
Sex, n (%)			0.3 (1)^d^	.59
Male	93 (63.7)	98 (66.7)		
Female	53 (36.3)	49 (33.3)		
Ethnicity, n (%)			<0.001 (1)^d^	.99
Han Ethnicity	140 (95.9)	141 (95.9)		
Other	6 (4.1)	6 (4.1)		
Medical insurance, n (%)			0.7 (1)^d^	.41
UEI^e^ and URI	101 (69.2)	95 (64.6)		
CMS^f^ and others	45 (30.8)	52 (35.4)		
Educational background, n (%)			0.2 (1)^d^	.68
≤Primary school	66 (45.2)	70 (47.6)		
≥Junior high school	80 (54.8)	77 (52.4)		
Employment status, n (%)			1.6 (1)^d^	.21
Employed and others	110 (75.3)	101 (68.7)		
Retired	36 (24.7)	46 (31.3)		
Marital status, n (%)			1.4 (1)^d^	.24
Married	130 (89)	124 (84.3)		
Others	16 (11)	23 (15.7)		
Cohabitation group, n (%)			5.0 (3)^d^	.17
Spouses living together	110 (75.3)	98 (66.7)		
Children living together	18 (12.3)	29 (19.7)		
Parents living together	10 (6.9)	7 (4.8)		
Living alone or other	8 (5.5)	13 (8.8)		
Individual income per month (RMB^g^), n (%)			0.8 (1)^d^	.33
≤2000	53 (36.3)	61 (41.5)		
>2000	93 (63.7)	86 (58.5)		
Smoking, n (%)			2.2 (1)^d^	.14
Yes	67 (45.9)	55 (37.4)		
No	79 (54.1)	92 (62.6)		
Alcohol consumption, n (%)			0.3 (1)^d^	.60
Yes	63 (43.2)	59 (40.1)		
No	83 (58.9)	88 (59.9)		
Electronic health management, n (%)			1.9 (1)^d^	.17
Yes	60 (41.1)	49 (33.3)		
No	86 (58.9)	98 (66.7)		
Type 2 diabetes duration (y), mean (SD)	3.78 (4.42)	4.09 (4.37)	−0.559 (291)^c^	.55
Medication duration (y), mean (SD)	3.21 (4.45)	3.52 (4.20)	−0.617 (291)^c^	.54
Antihyperglycemic medications, n (%)			2.4 (3)^d^	.50
0	25 (17.1)	24 (16.3)		
1	28 (19.2)	39 (26.5)		
2	69 (47.3)	64 (43.6)		
≥3	24 (16.4)	20 (13.6)		
Antihypertensive medications, n (%)			3.8 (2)^d^	.25
0	128 (87.7)	135 (91.8)		
1	16 (11)	12 (8.2)		
≥2	2 (1.3)	0 (0)		
Lipid-lowering medications, n (%)			5.7 (2)^d^	.06
0	96 (65.8)	112 (76.2)		
1	50 (34.2)	34 (23.1)		
≥2	0 (0)	1 (0.7)		
Complications^h^, n (%)			1.3 (1)^d^	.26
Yes	38 (26)	47 (32)		
No	108 (74)	100 (68)		

a IG: intervention group.

b CG: control group.

c represents *t* test.

d represents chi-square values.

e UEI and URI: Urban employee insurance and residential insurance.

f CMS and others: Cooperative Medical Scheme and others.

g The exchange rate used was RMB 7.11=US $1.

h Complications: diabetes-related complications.

### Between-Group Comparisons at 12 Weeks

At follow-up, the intervention group demonstrated greater reductions in HbA_1c_ (adjusted mean difference −0.38, 95% CI −0.68 to −0.09; *P*=.01), FBG (adjusted mean difference −0.75, 95% CI −1.27 to −0.44; *P*<.001), weight (adjusted mean difference −0.84, 95% CI −1.16 to −0.07; *P*=.03), BMI (adjusted mean difference −0.38, 95% CI −0.65 to −0.11; *P*=.01), systolic blood pressure (SBP; adjusted mean difference −2.71, 95% CI −4.62 to −0.79; *P*=.01), and diastolic blood pressure (DBP; adjusted mean difference −2.92, 95% CI −4.47 to −1.37, *P*<.001), TC (adjusted mean difference −0.27, 95% CI −0.48 to −0.05; *P*=.02), compared with the control group (Table 4). Moreover, the proportion of participants with an HbA_1c_ reduction ≥0.5% was significantly higher than in the control group (70.54% vs 57.82%; *χ*^2^_1_=5.2; *P*=.02).

**Table 4.** Baseline and 12-week outcomes, within-group changes, and adjusted between-group comparisons in participants with type 2 diabetes (N=293).

Outcomes	Baseline, mean (SD)	Week 12, mean (SD)	Within-group^a^		Adjusted between-group^b^	
			Mean difference (95% CI)	*P* value	Mean difference (95% CI)	*P* value
HbA_1c_^c^^,^^d^ (%)					−0.38 (−0.68 to −0.09)	.01
IG^e^ (n=146)	8.19 (2.14)	6.76 (1.31)	−1.43 (−1.76 to −1.11)	<.001		
CG^f^ (n=147)	8.11 (1.89)	7.12 (1.47)	−0.99 (−1.28 to −0.69)	<.001		
FBG^g^ (mmol/L)					−0.75 (−1.27 to −0.44)	<.001
IG (n=146)	8.05 (3.23)	6.44 (1.32)	−1.61 (−2.08 to −1.14)	<.001		
CG (n=147)	8.43 (3.06)	7.29 (2.18)	−1.14 (−1.61 to −0.67)	<.001		
Weight (kg)					−0.84 (−1.61 to −0.07)	.03
IG (n=146)	66.57 (13.38)	64.79 (12.55)	−1.78 (−2.41 to −1.15)	<.001		
CG (n=147)	66.18 (11.07)	65.17 (10.74)	−1.01 (−1.53 to −0.49)	<.001		
BMI (kg/m^2^)					−0.38 (−0.65 to −0.11)	.01
IG (n=146)	24.32 (3.35)	23.64 (3.10)	−0.68 (−0.90 to −0.47)	<.001		
CG (n=147)	24.47 (3.30)	24.12 (3.24)	−0.35 (−0.53 to −0.16)	<.001		
WC^h^ (cm)					−0.44 (−1.19 to 0.31)	.25
IG (n=146)	85.90 (10.23)	84.51 (9.68)	−1.39 (−1.98 to −0.80)	<.001		
CG (n=147)	86.57 (8.83)	85.48 (8.50)	−1.09 (−1.62 to −0.57)	<.001		
SBP^i^ (mm Hg)					−2.71 (−4.62 to −0.79)	.01
IG (n=146)	126.21 (16.48)	123.94 (12.41)	−2.27 (−3.98 to −0.56)	.01		
CG (n=147)	130.45 (15.87)	128.99 (13.33)	−1.46 (−3.16 to 0.25)	.09		
DBP^j^ (mm Hg)					−2.92 (−4.47 to −1.37)	<.001
IG (n=146)	78.05 (11.35)	75.95 (8.83)	−2.11 (−3.32 to −0.90)	.001		
CG (n=147)	80.31 (10.31)	79.98 (9.96)	−0.33 (−1.73 to 1.06)	.64		
TC^k^ (mmol/L)					−0.27 (−0.48 to −0.05)	.02
IG (n=146)	4.82 (1.41)	4.19 (1.26)	−0.63 (−0.84 to −0.43)	<.001		
CG (n=147)	4.66 (1.47)	4.33 (1.27)	−0.33 (−0.49 to −0.17)	<.001		
TG^l^ (mmol/L)					−0.18 (−0.41 to 0.05)	.12
IG (n=146)	2.70 (2.43)	2.18 (1.53)	−0.52 (−0.80 to −0.25)	<.001		
CG (n=147)	2.55 (1.73)	2.27 (1.37)	−0.28 (−0.46 to −0.10)	.003		
HDL^m^ (mmol/L)					0.08 (−0.01 to 0.16)	.08
IG (n=146)	1.28 (0.65)	1.42 (0.55)	0.14 (0.05 to 0.22)	.002		
CG (n=147)	1.24 (0.37)	1.33 (0.39)	0.08 (0.03 to 0.13)	.001		
LDL^n^ (mmol/L)					−0.16 (−0.34 to 0.02)	.08
IG (n=146)	2.85 (1.05)	2.53 (1.05)	−0.32 (−0.48 to −0.16)	<.001		
CG (n=147)	2.97 (1.02)	2.74 (1.01)	−0.22 (−0.35 to −0.10)	<.001		
Behaviors^o^					5.90 (3.67 to 8.14)	<.001
IG (n=146)	34.10 (12.76)	49.13 (10.45)	15.03 (12.41 to 17.65)	<.001		
CG (n=147)	34.30 (14.19)	43.66 (9.80)	9.36 (7.03 to 11.69)	<.001		
Self‐efficacy					2.90 (1.79 to 4.01)	<.001
IG (n=146)	25.42 (5.41)	31.28 (4.75)	5.86 (4.74 to 7.00)	<.001		
CG (n=147)	24.47 (5.52)	28.23 (5.09)	3.76 (2.78 to 4.75)	<.001		
Habit Strength					6.34 (4.32 to 8.37)	<.001
IG (n=146)	57.86 (13.08)	73.79 (8.81)	15.93 (13.69 to 18.16)	<.001		
CG (n=147)	55.78 (12.45)	67.03 (10.04)	11.26 (9.31 to 13.21)	<.001		

a Within-group mean differences represent changes from baseline to week 12.

b Adjusted between-group mean differences were estimated using ANCOVA, with baseline values and study center included as covariates.

c Primary outcome.

d HbA_1c_: hemoglobin A_1c_.

e IG: intervention group.

f CG: control group.

g FBG: fasting blood glucose.

h WC: waist circumference.

i SBP: systolic blood pressure.

j DBP: diastolic blood pressure.

k TC: total cholesterol.

l TG: triglycerides.

m HDL: high‐density lipoprotein cholesterol.

n LDL: low‐density lipoprotein cholesterol.

o Behaviors: diabetes self‐management behaviors.

Compared with controls, the intervention group achieved greater improvements in self-management behaviors (adjusted mean difference 5.90, 95% CI 3.67‐8.14; *P*<.001), self-efficacy (adjusted mean difference 2.90, 95% CI 1.79‐4.01; *P*<.001), and habit strength (adjusted mean difference 6.34, 95% CI 4.32‐8.37; *P*<.001). No significant between-group differences were observed for WC, triglycerides, HDL, or LDL (Table 4). No severe hypoglycemia occurred or nonsignificant difference was observed among medications among 2 groups (Table S1 in Multimedia Appendix 1).

Sensitivity analyses adjusting for baseline values only yielded results that were largely consistent with the primary analyses adjusted for both baseline values and study center. The direction and statistical significance of the primary and secondary outcomes remained unchanged, with the exception of weight, for which the between-group difference was attenuated and no longer statistically significant (Table S2 in Multimedia Appendix 1).

### Within-Group Changes From Baseline to 12 Weeks

Both 2 groups showed significant improvements in HbA_1c_, FBG, weight, BMI, WC, and blood lipids of TC, triglycerides, HDL, and LDL levels from baseline to 12 weeks. Blood pressure of SBP and DBP improved significantly only in the intervention group, with no significant changes observed in the control group. From baseline to follow-up, both groups demonstrated significant gains in self-management behaviors, self-efficacy, and habit strength (Table 4).

## Discussion

### Principal Findings

This multicenter randomized trial demonstrated that the DSE-BN program improved HbA_1c_, FBG, weight, BMI, blood pressure, and TC compared with standard digital diabetes education. The intervention was also associated with improvements in diabetes self-management behaviors, self-efficacy, and habit strength, highlighting the potential value of an integrated digital structured education program incorporating behavioral nudge tools and complementary digital support features to simultaneously support reflective and automatic behavioral processes in diabetes self-management.

### Interpretation of Findings

The observed improvements in glycemic outcomes in the intervention group, including a mean reduction of 0.38% in HbA_1c_ and decreases in FBG, are consistent with previous evidence suggesting that app-based diabetes education may support glycemic control by facilitating lifestyle modification and self-management behaviors [32]. Although the absolute HbA_1c_ reduction was modest, a significantly greater proportion of participants in the intervention group achieved an HbA_1c_ reduction of ≥0.5% compared with the control group, indicating that the intervention was capable of producing clinically meaningful improvements in a subset of participants. It is important to note that the mean HbA_1c_ reduction observed in this study falls below the 0.5% threshold commonly considered clinically meaningful at the individual level [33], and the relatively short follow-up period limits inference regarding long-term glycemic benefit. Nevertheless, modest HbA_1c_ reductions have been shown to be relevant at the population level. For example, findings from the UK Prospective Diabetes Study suggest that each 1% decrease in HbA_1c_ is associated with substantial reductions in diabetes-related complications [34]. While extrapolation should be interpreted with caution, the findings of this study suggest that even small improvements achieved through low-intensity, scalable digital interventions may have public health relevance if maintained over time.

In contrast, pharmacological agents like glucagon-like peptide-1 receptor agonists typically achieve HbA_1c_ reductions exceeding 1.2% [35]. Improvements observed in the control group are consistent with evidence that digital interventions can enhance cognitive-behavioral processes and health literacy [36,37]. Notably, although the intervention group received a more streamlined educational curriculum than the control group, it achieved superior improvements across several metabolic and behavioral outcomes. This finding may reflect the added value of the integrated intervention package, which combined digital structured education with multiple behavioral support components, including instructional videos, interactive quizzes, preparatory and postmodule review questions with immediate feedback, peer interaction, automated reminders, rule-based tailored feedback, data-driven early-warning alerts, and home-based behavioral nudge tools. Together, these integrated features may have reinforced both intentional self-management and habit formation, thereby supporting long-term engagement beyond education alone. Importantly, no significant between-group differences were observed in the use of antihyperglycemic medications at the 12-week follow-up, and no severe hypoglycemic events were reported, supporting the safety of the intervention and indicating that the observed effects were unlikely to be driven by pharmacological changes.

The design of the DSE-BN program was informed by dual-process theory, which proposes that health behaviors are influenced by both reflective and automatic behavioral processes [38]. Rather than relying on education alone, the intervention integrated digital structured education with complementary digital support features and behavioral nudge tools to reinforce healthy behaviors in participants’ home environments. By simultaneously strengthening reflective processes through structured education and supporting automatic processes through home-based behavioral nudge tools and digital behavioral support, the intervention may have facilitated the transition from intentional self-management to more consistent daily habits. These behavioral changes may, in turn, have contributed to the observed improvements in weight, BMI, blood pressure, and TC through better adherence to physical activity, medication use, and healthy dietary behaviors [39]. Consistent with this interpretation, no significant between-group differences were observed in the use of antihypertensive or lipid-lowering medications at the 12-week follow-up, suggesting that these cardiometabolic benefits were also unlikely to be driven by medication changes. Although this trial evaluated the overall effectiveness of an integrated intervention package rather than the independent effects of individual components, the findings suggest that integrating behavioral nudge tools within a broader digital structured education program may provide additional benefits beyond education alone.

Psychological enhancements in self-efficacy, self-management, and habit strength echo previous research [40], with structured education addressing personal and environmental factors per social cognitive theory [41]. From the perspective of dual-process theory, the integrated intervention may have further strengthened automatic behavioral processes while reinforcing the effects of reflective learning [26,42]. This integration of reflective and automatic behavioral processes may have facilitated the transition from conscious goal-setting to more habitual self-management behaviors, providing a theory-informed approach to promoting long-term diabetes self-management [43,44]. Future studies should investigate the neural mechanisms underlying behavioral changes following this dual-process intervention and evaluate the relative contribution of individual intervention components [45].

Consistent with American Diabetes Association and European Association for the Study of Diabetes guidelines recommending HbA_1c_ levels below 7% to mitigate complications [46], this nonpharmacological intervention serves as an adjunct for patients near targets or in combination with medications, enhancing accessibility in real-world practice.

### Implications for Digital Health Practice

This multicenter randomized trial demonstrates that an integrated digital structured education program incorporating behavioral nudge tools and complementary behavioral support strategies can enhance diabetes self-management by simultaneously supporting reflective and automatic behavioral processes. The intervention was associated with improvements in HbA_1c_, weight, BMI, blood pressure, TC, self-management behaviors, self-efficacy, and habit strength, addressing common limitations of digital interventions such as declining engagement and suboptimal adherence.

By extending support into patients’ home environments, this low-cost, technology-enabled approach shows strong potential for scalable, sustainable deployment in routine diabetes care, particularly in resource-constrained settings. Its favorable safety profile and independence from pharmacological changes further support its feasibility as an adjunct to standard treatment. These findings underscore the potential value of integrated, theory-informed digital interventions that integrate reflective learning with support for automatic behavior formation, providing a scalable approach to sustainable diabetes self-management.

### Study Limitations

Several limitations of this study should be acknowledged. Blinding of participants was infeasible due to the intervention’s behavioral nature, which may have introduced expectation bias. Outcomes were assessed only at 12 weeks, limiting inference regarding the sustainability of intervention effects over the longer term. Accordingly, improvements observed across multiple secondary outcomes should be interpreted cautiously. This relatively short follow-up period may also partly explain the attenuation of the between-group difference in weight observed in sensitivity analyses, suggesting that weight-related outcomes—given their multifactorial determinants—may be more sensitive to model specification than glycemic and behavioral outcomes. Recruitment from a single Chinese city limited population diversity, and the educated, urban, insured sample may reduce generalizability, particularly regarding socioeconomic, rural-urban, and digital literacy disparities that could exacerbate exclusion. In addition, because this study evaluated the overall effectiveness of an integrated intervention package, it was not designed to isolate the effects of individual intervention components. Future dismantling studies are warranted to identify the active components and optimize the design of theory-informed digital behavioral interventions. Despite these limitations, the multicenter randomized design, standardized intervention delivery, and the theory-informed dual-process framework bolster internal validity. Future studies with longer follow-up and more diverse populations are warranted to confirm and extend these findings.

### Conclusions

This study demonstrates that an integrated digital structured education program incorporating behavioral nudge tools can improve glycemic control, weight, BMI, blood pressure, TC, and self-management in adults with type 2 diabetes. Beyond clinical outcomes, the intervention was associated with improvements in psychological and behavioral factors, including self-efficacy and habit formation, which are critical for supporting short- to medium-term adherence in chronic disease management. These findings suggest that an integrated digital structured education program incorporating behavioral nudge tools provides a scalable, theory-informed approach that bridges intentional behavior change and habit formation, with potential to support sustainable diabetes self-management in routine care.

## Supplementary material

10.2196/91407 Multimedia Appendix 1 Supplementary results.[Aff aff1 aff2]

10.2196/91407 Checklist 1 CONSORT checklist.

## Abbreviations

C-SED: Chinese version of the Self-Efficacy for Diabetes
CONSORT: Consolidated Standards of Reporting Trials
DBP: diastolic blood pressure
DSE-BN: digital structured education with behavioral nudge tools
FBG: fasting blood glucose
HbA_1c_: hemoglobin A_1c_.
HDL: high-density lipoprotein cholesterol
ICC: intraclass correlation coefficient
LDL: low-density lipoprotein cholesterol
PASS: Power Analysis and Sample Size
SBP: systolic blood pressure
SMASS: self-management support system
SRHI: Self-Report Habit Index
TC: total cholesterol
WC: waist circumference
