# Supplementary material for: Digital Structured Education With Behavioral Nudge Tools for Adults With Type 2 Diabetes: Multicenter Randomized Controlled Trial
Source: JMIR Mhealth Uhealth. 2026 Sep 1;14:e91407. doi: 10.2196/91407 (PMC13542806; doi:10.2196/91407)
Supplement: Supplementary file 1 [file mhealth-v14-e91407-s002.pdf]

The CONSORT reporting checklist

For checking that reports of randomised trials can be understood and used by everyone

> Note
>
> If you have not used a reporting guideline before, read about how and why to use them and check whether CONSORT is the most applicable reporting guideline for your work.
>
> Reporting guidelines are most useful when used early in research. When writing a manuscript or application, consider using the Full Guidance where you'll see explanations and examples for each item.
>
> After writing, demonstrate adherence by completing this checklist:
>
> 1. Specify where each item is described (see Note 1).
> 2. Cite this checklist (See Note 2).
> 3. Include your completed checklist as a supplement when submitting to a journal so that future readers can use it to find information.

	Item Description	Location (or reason for not reporting)
**Title and Abstract**		
1a. Title	Identification as a randomised trial.	Page 1, line 1-2
1b. Structured Abstract	Structured summary of the trial design, methods, results, and conclusions.	Page 1-2, line 4-49
**Open Science**		
2. Trial Registration	Name of trial registry, identifying number (with URL) and date of registration.	Page 3, line 51-52; Page 13, line 310-317
Protocol and statistical analysis plan	Where the trial protocol and statistical analysis plan can be accessed.	Page 13, line 311-312
4. Data sharing	Where and how the individual de-identified participant data (including data dictionary), statistical code and any other materials can be accessed.	Page 21, line 516-518
5. Funding and Conflicts of Interest		
5a. Funding	Sources of funding and other support (eg, supply of drugs), and role of funders in the design, conduct, analysis, and reporting of the trial.	Page 21, line 510-513
5b. Conflicts of interest	Financial and other conflicts of interest of the manuscript authors.	Page 21, line 514-515

**Introduction**		
6. Background and rationale	Scientific background and rationale.	Page 3-5, line 56-117
7. Objectives	Specific objectives related to benefits and harms.	Page 5, line 109-117
**Methods**		
8. Patient and public involvement	Details of patient or public involvement in the design, conduct and reporting of the trial.	Page 6-7, line134-150
9. Trial Design	Description of trial design including type of trial (eg, parallel group, crossover), allocation ratio, and framework (eg, superiority, equivalence, non-inferiority, exploratory).	Page 5-6, line 120-125
10. Changes to trial protocol	Important changes to the trial after it commenced including any outcomes or analyses that were not pre-specified, with reason.	Page 5-6, line 122-125
11. Trial Setting	Settings (eg, community, hospital) and locations (eg, countries, sites) where the trial was conducted.	Page 5-6, line 120-125
12. Eligibility Criteria		
12a. Participants	Eligibility criteria for participants.	Page 6-7, line 134-150
12b. Other	If applicable, eligibility criteria for sites and for individuals delivering the interventions (eg, surgeons, physiotherapists).	Not applicable
13. Intervention and comparator	Intervention and comparator with sufficient details to allow replication. If relevant, where additional materials describing the intervention and comparator (eg, intervention manual) can be accessed.	Page 8-10, table 1 and supplementary table 1
14. Outcomes	Prespecified primary and secondary outcomes, including the specific measurement variable (eg, systolic blood pressure), analysis metric (eg, change from baseline, final value, time to event), method of aggregation (eg, median, proportion), and time point for each outcome.	Page 11-12
15. Harms	How harms were defined and assessed (eg, systematically, non-systematically).	Page 12, line 18-23
16. Sample Size		
16a. How sample size was determined	How sample size was determined, including all assumptions supporting the sample size calculation.	Page 6-7, line 142-150
16b. Interim analyses and stopping criteria	Explanation of any interim analyses and stopping guidelines.	Page 6, line 129-130
17. Randomisation		
17a. Sequence	Who generated the random allocation sequence and	Page 7, line 151-163

Generation	the method used.	
17b. Type of Randomisation	Type of randomisation and details of any restriction (eg, stratification, blocking, and block size).	Page 7, line 151-163
18. Allocation concealment mechanism	Mechanism used to implement the random allocation sequence (eg, central computer/telephone; sequentially numbered, opaque, sealed containers), describing any steps to conceal the sequence until interventions were assigned.	Page 7, line 151-163
19. Implementation	Whether the personnel who enrolled and those who assigned participants to the interventions had access to the random allocation sequence.	Page 7, line 151-163
20. Blinding		
20a. Who was blinded	Who was blinded after assignment to interventions (eg, participants, care providers, outcome assessors, data analysts).	Page 7, line 151-163
20b. How blinding was achieved	If blinded, how blinding was achieved and description of the similarity of interventions.	Page 7, line 151-163
21. Statistical methods		
21a. Comparing groups	Statistical methods used to compare groups for primary and secondary outcomes, including harms.	Page 13-14, line 318-344
21b. Definition of who is included in each analysis	Definition of who is included in each analysis (e.g., all randomised participants), and in which group.	Page 13-14, line 318-344
21c. Missing Data	How missing data were handled in the analysis.	Page 13-14, line 318-344
21d. Additional Analyses	Methods for any additional analyses (eg, subgroup and sensitivity analyses), distinguishing pre-specified from post hoc.	Page 13-14, line 318-344
22. Participant flow, including flow diagram		
22a. Participant Numbers	For each group, the numbers of participants who were randomly assigned, received intended intervention, and were analysed for the primary outcome.	Page 14-15, line 345-351, figure 1
22b. Losses and exclusions	For each group, losses and exclusions after randomisation, together with reasons.	Page 14-15, line 345-351, figure 1
23. Recruitment		
23a. Dates	Dates defining the periods of recruitment and follow-up for outcomes of benefits and harms.	Page 6, line 126-130
23b. Reasons for stopping recruitment	If relevant, why the trial ended or was stopped.	Not applicable

24. Intervention and comparator delivery		
24a. As Administered	Intervention and comparator as they were actually administered (eg, where appropriate, who delivered the intervention/comparator, whether participants adhered, whether they were delivered as intended (fidelity)).	Page 7-9, line 164-179; Page 8-10, table 1 and supplementary table 1
24b. Concomitant Care	Concomitant care received during the trial for each group.	Page 11, line 248-250
25. Baseline Data	A table showing baseline demographic and clinical characteristics for each group.	Page 15, line 352-362, table 2 & 3
26. Numbers analysed, outcomes, and estimation	For each primary and secondary outcome, by group:<br>• the number of participants included in the analysis.<br>• the number of participants with available data at the outcome time point.<br>• result for each group, and the estimated effect size and its precision (such as 95% confidence interval).<br>• for binary outcomes, presentation of both absolute and relative effect size.	Page 15-16, table 4
27. Harms	All harms or unintended events in each group.	Page 16, line 378-380
28. Ancillary Analyses	Any other analyses performed, including subgroup and sensitivity analyses, distinguishing pre-specified from post hoc.	Page 16, line 373-380
**Discussion**		
29. Interpretation	Interpretation consistent with results, balancing benefits and harms, and considering other relevant evidence.	Page 16-21, line 396-506
30. Limitations	Trial limitations, addressing sources of potential bias, imprecision, generalisability, and, if relevant, multiplicity of analyses.	Page 19-20,- line 465-481

1. Hopewell S, Chan AW, Collins GS, Hróbjartsson A, Moher D, Schulz KF, et al. CONSORT 2025 statement: Updated guideline for reporting randomised trials. The BMJ [Internet]. 2025 Apr;389:e081123. Available from: https://www.ncbi.nlm.nih.gov/pmc/articles/PMC11995449/

2. Hopewell S, Chan AW, Collins GS, Hróbjartsson A, Moher D, Schulz KF, et al. The CONSORT reporting checklist. In: Harwood J, Albury C, Beyer J de, Schlüssel M, Collins G, editors. The EQUATOR network reporting guideline platform [Internet]. The UK EQUATOR Centre; 2025. Available from: https:/resources.equator-network.org/reporting-guidelines/consort/consort-checklist.docx
