# Supplementary material for: Digital Structured Education With Behavioral Nudge Tools for Adults With Type 2 Diabetes: Multicenter Randomized Controlled Trial
Source: JMIR Mhealth Uhealth. 2026 Sep 1;14:e91407. doi: 10.2196/91407 (PMC13542806; doi:10.2196/91407)
Supplement: Supplementary file 2 [file mhealth-v14-e91407-s001.docx]

**Table S1. Medications in patients with type 2 diabetes mellitus by group at 12-week follow-up** (N = 293)

| **Oral medication** | **IG (n=146)**  **n (%)** | **CG (n=147)**  **n (%)** | ***χ^2^*** | ***P*** |
| --- | --- | --- | --- | --- |
| Antihyperglycemic medications |  |  | 0.912 | .82 |
| 0 | 23 (15.8) | 22 (15.0) |  |  |
| 1 | 34 (23.3) | 41 (27.9) |  |  |
| 2 | 66 (45.2) | 64 (43.5) |  |  |
| ≥3 | 23 (15.8) | 20 (13.6) |  |  |
| Antihypertensive medications |  |  |  |  |
| 0 | 135 (92.5) | 135 (91.8) | 0.044 | .98 |
| 1 | 10 (6.8) | 11 (7.5) |  |  |
| ≥2 | 1 (0.7) | 1 (0.7) |  |  |
| Antilipidemic agents |  |  | 3.321 | .07 |
| 0 | 95 (65.1) | 110 (74.8) |  |  |
| 1 | 51 (34.9) | 37 (25.2) |  |  |

Note: IG, intervention group; CG, control group.

**Table S2. Sensitivity analysis of between-group outcomes at 12 weeks adjusted for baseline values** **(N = 293)**

| **Outcomes** | **IG (n=146)**  **Mean±SD** | **CG (n=147)**  **Mean±SD** | **Mean difference [95%CI]** | ***P*** |
| --- | --- | --- | --- | --- |
| *HbA1c, % | 6.76±1.31 | 7.12±1.47 | -0.38 (-0.68, -0.09) | .01 |
| FBG, mmol/L | 6.44±1.32 | 7.29±2.18 | -0.76 (-1.13, -0.38) | ＜.001 |
| Weight, kg | 64.79±12.55 | 65.17±10.74 | -0.738 (-1.51, 0.04) | .06 |
| BMI, kg/m^2^ | 23.64±3.10 | 24.12±3.24 | -0.35 (-0.62, -0.09) | .01 |
| WC, cm | 84.51±9.68 | 85.48±8.50 | -0.37 (-1.12, 0.38) | .33 |
| SBP, mmHg | 123.94±12.41 | 128.99±13.33 | -2.5 (-4.41, -0.54) | .01 |
| DBP, mmHg | 75.95±8.83 | 79.98±9.96 | -67 (-4.23, -1.11) | ＜.001 |
| TC, mmol/L | 4.19±1.26 | 4.33±1.27 | -0.24 (-0.46, -0.02) | .03 |
| TG, mmol/L | 2.18±1.53 | 2.27±1.37 | -0.17 (-0.40, 0.05) | .14 |
| HDL, mmol/L | 1.42±0.55 | 1.33±0.39 | 0.07 (-0.02, 0.15) | .11 |
| LDL, mmol/L | 2.53±1.05 | 2.74±1.01 | -0.14 (-0.32, 0.05) | .14 |
| Behaviors | 49.13±10.45 | 43.66±9.80 | 5.50 (3.21, 7.79) | ＜.001 |
| Self‐efficacy | 31.28±4.75 | 28.23±5.09 | 2.89 (1.75, 3.97) | ＜.001 |
| Habit Strength | 73.79±8.81 | 67.03±10.04 | 6.19 (4.16, 8.23) | ＜.001 |

Note: *Primary outcome. IG, intervention group; CG, control group; HbA1c, Glycated Hemoglobin A1c; FBG, Fasting Blood Glucose; BMI, body mass index; WC, waist circumstance; SBP, systolic blood pressure; DBP, diastolic blood pressure; TC, total cholesterol; TG, triglycerides; HDL, high‐density lipoprotein cholesterol; LDL, low‐density lipoprotein cholesterol; Behaviors, diabetes self‐management behaviors.
